# Case report - Adrenal collision tumour composed of oncocytoma and pheochromocytoma

**DOI:** 10.3389/fonc.2025.1554355

**Published:** 2025-08-08

**Authors:** Jovana Lukic, Sanja Ilic, Rastko Zivic, Stefan Mitic, Predrag Savic, Vladan Vukomanovic, Milos Petrovic

**Affiliations:** ^1^ Clinic for Internal Medicine, Clinical Hospital Centre “Dragisa Misovic - Dedinje”, Belgrade, Serbia; ^2^ Clinic for Surgery, Clinical Hospital Centre “Dragisa Misovic – Dedinje”, Belgrade, Serbia; ^3^ School of Medicine, University of Belgrade, Belgrade, Serbia

**Keywords:** adrenal collision tumour, oncocytoma, pheochromocytoma, adrenal surgery, paraganglioma

## Abstract

Adrenal collision tumours (ACTs) are rare clinical entities denoting separate coexisting tumours involving adrenal glands. Here, we report the clinical, radiological and pathohistological presentation of a 49-year-old patient with an ACT composed of oncocytoma and pheochromocytoma. Following the initial diagnostic procedure guided by suspicion of pheochromocytoma, the patient has undergone surgery, recovered well and has been followed since. In the resected mass, oncocytoma was an incidental finding, as is typical of this type of tumour. With both components of this particular ACT being rare, this is a reminder of a need for widening differential diagnostic options when evaluating the patient for adrenal masses of unknown origin.

## Introduction

Adrenal collision tumours (ACTs) are rare clinical entities denoting separate coexisting tumours involving adrenal glands, with the sharp demarcation between the two and without a substantial histological admixture at the interface (1, 2). ACTs may exist as a combination of benign/benign, benign/malignant or malignant/malignant tumours. The actual prevalence of ACTs is unknown, as many may go undetected because of the small size of one component and/or the sampling error. They commonly consist of adrenal cortical adenoma, pheochromocytoma or a metastatic malignant tumour (2, 3). The most commonly described collision tumour in the adrenal gland is a cortical adenoma with myelolipoma. To the best of our knowledge, a collision tumour of pheochromocytoma with oncocytoma has not previously been reported in the literature (1, 2, 4).

## Case description

A 49-year-old female patient has been admitted to the department of endocrinology, providing anamnestic data of the episodes of arterial hypertension occurring once or twice a month in the last 3 years, reaching 170/100 mmHg, manifesting as occipital headaches and dizziness, accompanied by sweating and palpitations. Several years prior, the patient was treated in the cardiology department for high blood pressure, but did not respond to therapy. The patient was a non-smoker and the medical history was negative. Family history was similarly inconspicuous. General state at the admission was normal (arterial pressure 140/90 mmHg, glucose 6 mmol/L, ECG sinus rhythm, frequency 90/min, without ST/T changes). Physical examination revealed no palpable flank masses or tenderness.

**Figure 1:**
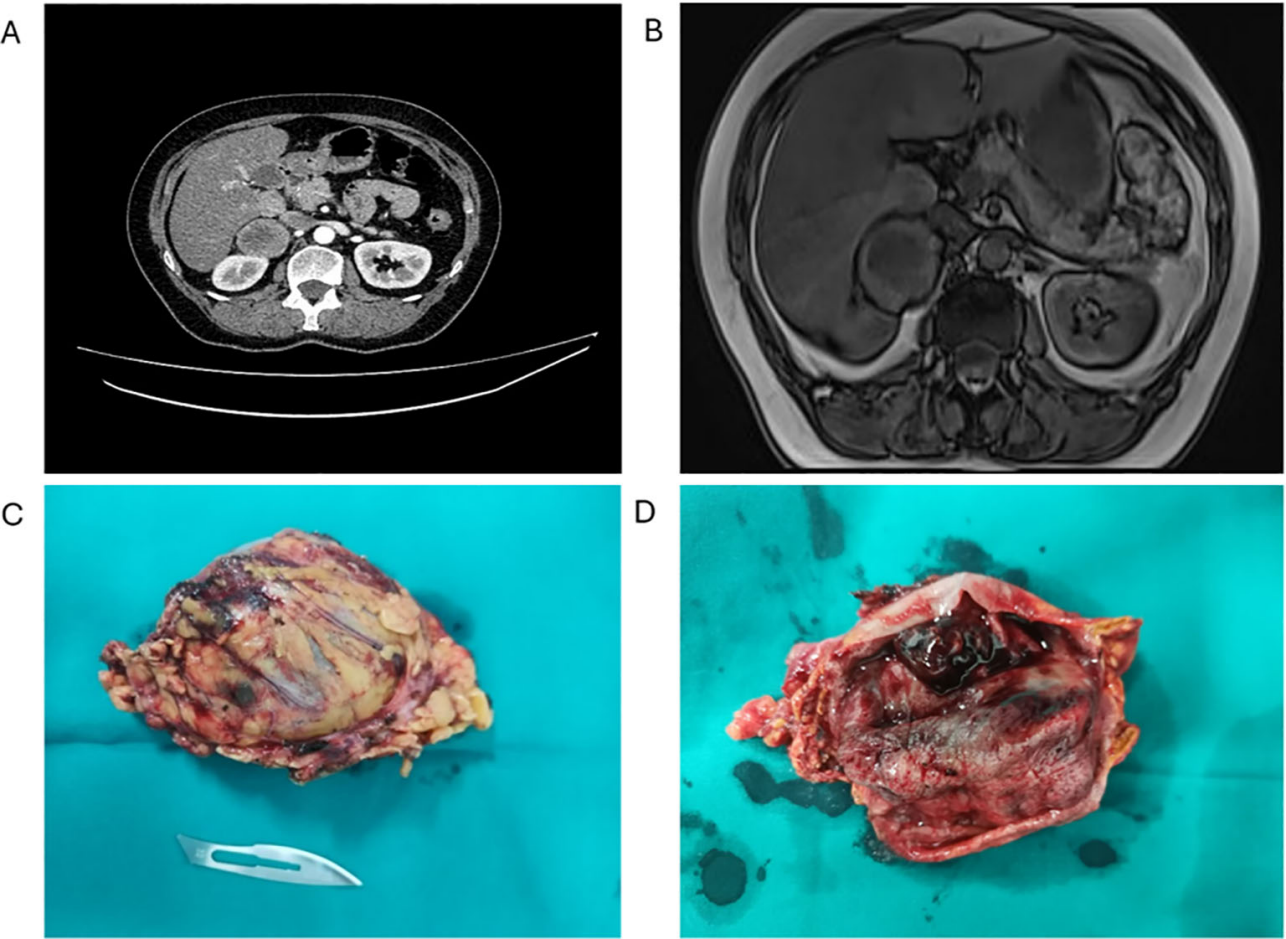
Preoperative CT scan and macroscopic appearance of the tumour. **(A)** Abdominal CT showing an expansive solid cystic-necrotic mass (arrow) in the cavity of the right adrenal gland, 5.5 x 5.7 cm in size, with continuity in the right lobe of the liver without infiltration, posteriorly pushing into the kidney. **(B)** Abdominal MRI showing a cystic mass indicative of a necrotic tumour, having a wall of irregular thickness, pushing into the right lobe of the liver with signs of possible infiltration (arrow). **(C)** Macroscopic appearance of the ACT (81 X 49 x 38 mm) after adrenalectomy. **(D)** The tumour was well-defined, round and encapsulated, with characteristic brown cross-section.

Abdominal and pelvic ultrasound examination showed a cystic tumour in the cavity of the right adrenal gland, hyperechogenic with weak CDS signal (70 mm x 54 mm). This, in combination with the clinical symptoms, prompted the diagnostic search in the direction of intra-adrenal sympathetic paraganglioma (pheochromocytoma), for which the arterial hypertension resistant to therapy may be a predominant sign (5). Further diagnostics has been performed after the patient was admitted, including 24h ambulatory blood pressure monitoring, with mean BP values being similar both while awake and asleep (131 ± 13/78 ± 10 mmHg and 139 ± 19/76 ± 10 mmHg, respectively). Laboratory analysis showed hyperglycaemia without DM, as another finding indicative of intra-adrenal sympathetic paraganglioma (5), with the levels of metanephrine 239.8 pg/L, normetanephrine 3003.7 pg/L and chromogranin A 309.8 µg/L (samples tested twice for confirmation). Other parameters of adrenal and thyroid function were within reference values (**Table 1**). Additionally, PTH and calcitonin levels in this patient were checked to rule out MEN 2 Syndromes, both being within their respective reference ranges (PTH 59.5 [18.5-88.0] pg/mL, calcitonin 7.2 [< 13.8] pg/ml]). We have also performed the calcium stimulation test, which also indicated normal function. In the abdominal CT scan, a tumour mass (55x46x77 mm) in the right suprarenal cavity was verified, having both solid and cystic structure, combined with the areas of necrosis, without the signs of infiltration in the hepatic parenchyma. There were no signs of intravascular infiltration (**Figure 1A**) either.

**Table 1:** Laboratory analyses, showing highly elevated markers for pheochromocytoma, marked with *.

Analysis	Result	Units	Reference values	Methods
Metanephrine, free in plasma	239.8 *	pg/ml	<65	ELISA
Chromogranine A	309.8*	µg/ml	<100	ELISA
Normetanephrine, free in plasma	3003.7*	pg/l	<196	ELISA
FT3	3.6	pmol/L	2.9-4.9	
FT4	11.26	pmol/l	9.01-19.05	
TSH	0.399	mU/l	0.35-4.94	
PTH	59.5	pg/mL	18.5-88.0	
Calcitonin	7.2	pg/ml	< 13.8	
FSH	73.3	iU/l	23-116,3 postmenopause	
LH	51.1	iU/l	>30 postmenopause	
PR	1.4	nmol/l	<2.3 postmenopause	
CORT	563	nmol/l	145-619	

After the CT scan, MRI confirmed a nodular tumour, raising a suspicion of the propagation of the primary process and the infiltration of the surrounding organs (**Figure 1B**). The heterogeneous nature of the tumour mass implied the presence of two distinct neoplastic processes. As magnetic resonance imaging (MRI) and multidetector computed tomography imaging techniques may describe different tumour components separately, a biopsy may be required in selected patients for confirmation (2). Importantly, no local or distant metastases were observed in the abdomen or pelvis.

Therapy for this kind of tumour is surgical and the surgical technique can be laparoscopic or open, depending on the tumour anatomy and the experience of the surgeon. A multidisciplinary evaluation was performed and, considering that the patient was seeking a definitive treatment option, she consented to a right open adrenalectomy. Preoperative preparation was conducted for the duration of 14 days before surgery with alpha-(phenoxybenzamine) and beta- (bisoprolol) adrenergic blockers.

The extracted tumour mass was round, solid, well-defined, with characteristically dark cross-section and the areas of necrosis (**Figure 1C**). As the MRI indicated possible infiltration of the liver, it was biopsied, but instead of the tumour, the presence of cirrhosis was established later in the pathohistology examination (**Figure 1D**). There were no complications in the postoperative period and the patient was discharged in good condition. She is having regular check-ups with her endocrinologist and, since the surgery, no additional therapy was needed.

**Figure 2:**
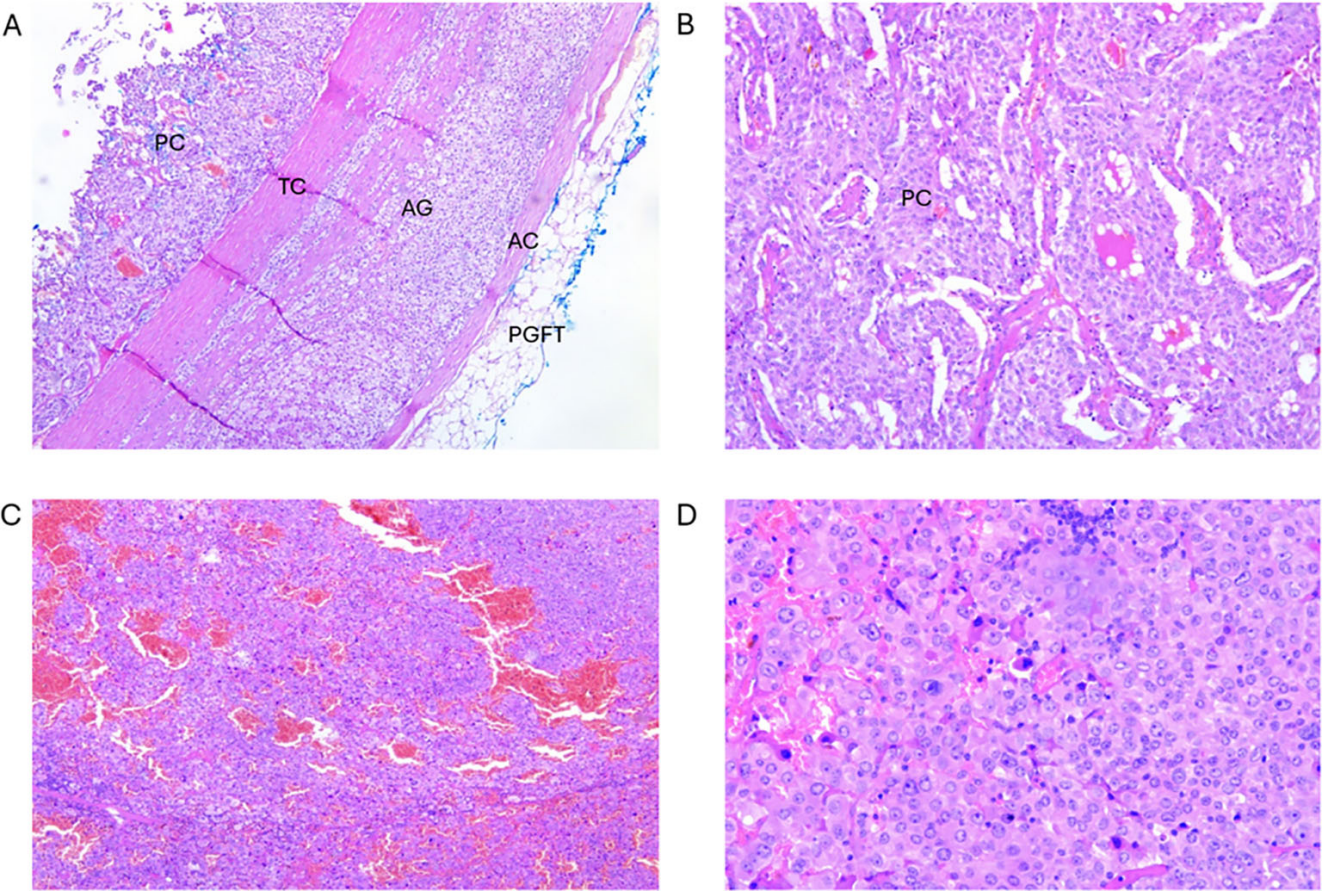
Tumour I: **(A)** Cross-section of pheochromocytoma component of the ACT. PC- pheochromocytoma; TC, tumour capsule; AG, adrenal gland; AC, adrenal capsule; PGFT, periglandular fat tissue (magnification 50x). **(B)** Pheochromocytoma component enlarged from A (magnification 100x). Tumour II: **(C)** Cross-section of oncocytoma component of the ACT (magnification 50x). **(D)** Enlarged section from C (magnification 200x).

Pathohistological assessment confirmed the presence of pheochromocytoma, as expected, while the other component was diagnosed as adrenal oncocytoma (**Figures 2A, B**). The latter component was composed of the cells with a typical “oncocytic” cell morphology (**Figures 2C, D**), i.e. having large polygonal cells with eosinophilic granular cytoplasm. Altogether, given the presence of two adjacent tumour components, the final diagnosis was that of an ACT.

To assess the potential for malignancy, individual components were further analysed using their respective scoring systems: pheochromocytoma was assessed against PASS, whereas oncocytoma component was analysed according to Lin-Weiss-Bisceglia scoring system (**Table 2**).

**Table 2:** Scoring scales for pheochromocytoma (PASS) and oncocytoma (LWB).

Pheochromocytoma of the adrenal gland scoring scale (PASS):		
Histomorphological parameter	Score	Score if present (no. of points assigned)
Large nests or diffuse growth (*>*10% of tumour volume)	2	2
Central or confluent tumour necrosis	2	2
High cellularity	1	2
Cellular monotony	0	2
Tumour cel spindling (even if focal)	0	2
More than 3 mitotic figures/10 high-power field	2	2
Atypical mitotic figures	0	2
Extension into adipose tissue	0	2
Vascular invasion	0	1
Capsular invasion	1	1
Profound nuclear pleomorphism	0	1
Nuclear hyperchromasia	0	1
**Total**	**8**	**20**
Modified Lin-Weiss-Bisceglia (LWB) scoring system for oncocytic adrenal neoplasms:		
1) Major criteria:		
High mitotic rate (> 5 mitoses/50 HPF)		Yes
Atypical mitotic figures		No
Venous invasion		No
2) Minor criteria:		
Large size (> 10 cm) or weight (> 200 g)		No
Necrosis		No
Sinusoidal invasion		No
Capsular invasion		No

Our patient’s PASS score was 8, indicating a malignancy risk, whereas with regards to the Lin-Weiss-Bisceglia (LWB) scoring system, only high mitotic rate (> 5 mitoses/50 HPF) was present among the major criteria (and none within the minor ones).

## Discussion

Collision tumours can occur in various organs such as the lungs, liver, and genitourinary tract. An ACT is an infrequently described tumour entity, comprising two different neoplasms that coexist adjacent to one another within a single adrenal mass. Each ACT is given a name based on the cell types within that particular tumour (2). A retrospective review of adrenalectomy specimens has estimated ACTs to comprise approximately 1–4% of adrenal tumours, although precise epidemiological data are lacking. They are more frequently identified during histopathological examination following adrenalectomy for presumed single lesions. The most frequent combinations involve adrenal adenomas with myelolipomas, metastases, or pheochromocytomas. Adrenal oncocytomas themselves are rare, with less than 300 cases reported globally (6). The pathogenesis of the ACTs is unclear. Various mechanisms have been postulated, including a single carcinogenic stimulus altering a particular region of the adrenal gland, allowing two separate tumours to develop in close proximity; another explanation is the presence of one tumour altering the local environment, providing a fertile ground for the development of the other tumour (2, 4).

Our patient’s ACT had two components, oncocytoma and pheochromocytoma. The preoperative assumption of the presence of pheochromocytoma was consistent with clinical manifestations, laboratory data and imaging results. The presence of pheochromocytoma in our patient was mostly manifested by occasional bouts of arterial hypertension and palpitations. Importantly, intratumoural necrosis, relative preponderance of fibrotic interstitium compared to chromaffin cells or the impeded release of the catecholamines due to encapsulation of the tumour by the connective tissue may lead to such paucity of symptoms.

Due to the size (>6 cm) of the tumour and considering that minimal manipulation and speedy removal are essential to reduce the release of catecholamines, open surgery was advocated. This was further supported by the CT/MRI finding of the intra-tumoural necrosis, which increases the probability of rupture during surgical handling. Over 90% of adrenal tumors are being operated laparoscopically (lateral, transperitoneal) in our institution, but because the MRI indicated possible infiltration of the liver, we decided to perform open surgery. Although laparoscopic adrenalectomy is standard for benign and smaller adrenal lesions, open surgery is often preferred when there is suspicion of malignancy or risk of capsular disruption. Additionally, surgical access can be achieved via various approaches including transperitoneal, retroperitoneal, posterior or lateral techniques, each with specific advantages depending on the tumour location, size and surgeon expertise. The transperitoneal approach provides wide exposure and is preferred for large or invasive tumours, while the retroperitoneal approach may be favoured for smaller, posteriorly located tumours due to its minimally invasive nature. Selection of the surgical method must be individualised based on tumour characteristics, anatomical considerations and institutional experience (7–11).

Given the choice of therapy, preoperative preparation was obligatory, because during the surgical manipulation of the tumour, dangerous amounts of catecholamines can be released into the circulation, causing life-threatening events (12). The main goals of preoperative management of a pheochromocytoma patient are to normalise blood pressure and heart rate, restore volume depletion and prevent a catecholamine storm and consequent haemodynamic instability during the surgery (13). Phenoxybenzamine is the non-selective alpha-blocker most widely used to prevent complications during the surgery. Beta blockers are not frequently used preoperatively in this setting, unless for the control of tachycardia, as was the case with our patient (14, 15).

After the surgery, as expected, pathohistological assessment confirmed the presence of intra-adrenal sympathetic paraganglioma (i.e. pheochromocytoma), with the incidental finding of oncocytoma, thus forming a diagnosis of a rare collision tumour (**Figure 2**).

Pheochromocytoma or intra-adrenal sympathetic paraganglioma is a tumour of chromaffin cells of adrenal medulla, linked to a production of catecholamines with characteristically increased concentrations of metanephrine and normetanephrine in urine and plasma (16). They mostly develop in the 3-5^th^ decade of life, having an equal distribution in men and women, with up to 25% of them being malignant (5). Some of the rare examples in the literature of collision tumours that contain one of these two tumours are a case of coexisting oncocytoma and ganglioneuroma in the same adrenal gland and a case of adrenocortical adenoma and pheochromocytoma (3, 17).

When applied to our patient’s tumour, Pheochromocytoma of the Adrenal Gland Scaled Score (18) (PASS = 8) indicated a possible malignancy (19) (**Table 2**). However, it is important to emphasise that the Pheochromocytoma of the Adrenal gland Scaled Score (PASS) is designed to predict the risk of malignant behaviour in pheochromocytomas based on histological features, with score ≥4 indicating increased malignant potential. Indeed, our patient’s tumour did manifest histological features indicating potential malignancy and yet, the only absolute criterion of malignancy is evidence of the metastatic tumour spread, which was not proven (20). Importantly, if a mixed benign and malignant collision tumour is biopsied and only the benign component is identified, the suboptimal treatment may be delivered, with a significant impact on prognosis (4).

Following resection, ongoing surveillance is crucial. This includes regular clinical evaluation, biochemical screening (plasma/urinary metanephrines) and periodic imaging to monitor for recurrence or late metastasis. Although it is now recognised that a significant proportion of pheochromocytomas may be hereditary, the family history of our patient rather indicated a sporadic occurrence, possibly by a germline mutation in one of the known susceptibility genes (21–23). Similarly, there was no indication of syndromic association (e.g. MEN2) (24). Importantly, emerging literature supports routine genetic testing for pheochromocytoma, even in the absence of syndromic features or family history, as up to 40% may harbour germline mutations. While our patient showed no features suggestive of MEN2 or other hereditary syndromes, such testing should still be considered to guide long-term management and familial screening.

Oncocytomas most frequently develop within the kidney, as well as in parathyroid, pituitary, thyroid and salivary glands (25). Oncocytomas of the adrenal gland are, however, quite uncommon. Most of them are benign and nonfunctioning, with around 30 cases described, including a single report of a malignant functioning adrenocortical oncocytoma (26, 27). So far, less than 300 occurrences were recorded (6). The previous reports show that they occur more frequently in females (2.5:1), and on the left side (3.5:1) (25). Thus, although our patient was female, the sidedness of the oncocytoma was less than usual, possibly due to its association with pheochromocytoma in the ACT. Similarly, around 70% are non-functional in terms of adrenocortical hormone production, as was the case with our patient. As the CT and MRI features of oncocytomas are non-specific, distinguishing them from other adrenal neoplasms is diagnostically challenging. Thus, histological verification is ultimately necessary.

To assess the malignant potential of these rare tumours, Lin-Weiss-Bisceglia (LWB) system was put up as a refinement of general Weiss criteria (28) and is now accepted as a validated tool (29). LWB system consists of major and minor criteria. Presence of any of the major criteria indicates malignancy, the presence of any of the minor criteria is indicative of the borderline or uncertain malignant potential, while if none of major or minor criteria are present, the neoplasm would be considered benign.

Histologically, oncocytomas are characterised by a distinctive granular cytoplasmic eosinophilia of the large neoplastic cells. These cells are called oncocytes because of the “swollen” appearance they have as the result of a striking accumulation of mitochondria, occupying up to the 60% of the cytoplasm (4, 30) (**Figures 2C, D**), possibly associated with the finding that oncocytomas may result from the mutations in the mitochondrial DNA (31).

The short-term outcome for our patient has been excellent, with normalisation of blood pressure and no recurrence observed during follow-up. Nevertheless, due to the elevated PASS score, long-term surveillance is warranted. One significant limitation is the lack of genetic testing, which precludes a complete assessment of hereditary predisposition.

## Conclusion

To our knowledge, here we present the first case of a patient with the adrenal collision tumour comprising pheochromocytoma and oncocytoma. On their own, both occur rarely – thus, their combination presented here is a reminder to the clinicians to consider even such unusual variants in their diagnostic thinking.

## Data Availability

The raw data supporting the conclusions of this article will be made available by the authors, without undue reservation.

## References

[1] Foresti M Parmiggiani A. Adrenal adenoma-hemangioma collision tumor: description of two cases. J Radiol Case Rep. (2019) 13:1–12. doi: 10.3941/jrcr.v13i6.3691, PMID: 31558958 PMC6742450

[2] Katabathina VS Flaherty E Kaza R Ojili V Chintapalli KN Prasad SR. Adrenal collision tumors and their mimics: multimodality imaging findings. Cancer Imaging. (2013) 13:602–10. doi: 10.1102/1470-7330.2013.0053, PMID: 24434021 PMC3893905

[3] Lee HS Choi YJ Kim C Kim BH. Adrenal collision tumor: coexistence of pigmented adrenal cortical oncocytoma and ganglioneuroma. Case Rep Surg. (2016) 2016:5790645. doi: 10.1155/2016/5790645, PMID: 28053800 PMC5178330

[4] Liu D Kumar SA. An exceedingly rare adrenal collision tumor: adrenal adenoma-metastatic breast cancer-myelolipoma. J Community Hosp Intern Med Perspect. (2017) 7:241–4. doi: 10.1080/20009666.2017.1362315, PMID: 29046752 PMC5637651

[5] Farrugia FA Charalampopoulos A. Pheochromocytoma. Endocr Regul. (2019) 53:191–212. doi: 10.2478/enr-2019-0020, PMID: 31517632

[6] Coppola Bottazzi E Gambardella C Mongardini FM Vanella S Noviello A Palma T. Prognosis of adrenal oncocytic neoplasms (AONs): literature review of 287 cases and presentation of the oldest patient. J Clin Med. (2023) 12:6925. doi: 10.3390/jcm12216925, PMID: 37959390 PMC10649738

[7] Flávio Rocha M Faramarzi-Roques R Tauzin-Fin P Vallee V Leitao de Vasconcelos PR Ballanger P. Laparoscopic surgery for pheochromocytoma. Eur Urol. (2004) 45:226–32. doi: 10.1016/j.eururo.2003.09.016, PMID: 14734011

[8] Fernández-Cruz L Sáenz A Taurá P Sabater L Astudillo E Fontanals J. Helium and carbon dioxide pneumoperitoneum in patients with pheochromocytoma undergoing laparoscopic adrenalectomy. World J Surg. (1998) 22:1250–5. doi: 10.1007/s002689900554, PMID: 9841753

[9] Marty J Desmonts JM Chalaux G Fischler M Michon F Mazze RI. Hypertensive responses during operation for phaeochromocytoma: a study of plasma catecholamine and haemodynamic changes. Eur J Anaesthesiol. (1985) 2:257–64., PMID: 4065104

[10] Carter YM Mazeh H Sippel RS Chen H. Safety and feasibility of laparoscopic resection for large (≥ 6 CM) pheochromocytomas without suspected Malignancy. Endocr Pract. (2012) 18:720–6. doi: 10.4158/EP12014.OR, PMID: 22982788 PMC3468692

[11] Conzo G Pasquali D Della Pietra C Napolitano S Esposito D Iorio S. Laparoscopic adrenal surgery: ten-year experience in a single institution. BMC Surg. (2013) 13 Suppl 2:S5. doi: 10.1186/1471-2482-13-S2-S5, PMID: 24267584 PMC3850966

[12] Munakomi S Rajbanshi S Adhikary PS. Case Report: A giant but silent adrenal pheochromocytoma - a rare entity. F1000Res. (2016) 5:290. doi: 10.12688/f1000research, PMID: 27785358 PMC5022706

[13] Pacak K. Preoperative management of the pheochromocytoma patient. J Clin Endocrinol Metab. (2007) 92:4069–79. doi: 10.1210/jc.2007-1720, PMID: 17989126

[14] Tian J Bao Z Yuan Y Fang D Zhan Y Wang T. The duration of preoperative administration of single α-receptor blocker phenoxybenzamine before adrenalectomy for pheochromocytoma: 18 years of clinical experience from nationwide high-volume center. BioMed Res Int. (2019) 2019:2613137. doi: 10.1155/2019/2613137, PMID: 31828097 PMC6881764

[15] Lenders JWM Eisenhofer G Mannelli M Pacak K. Phaeochromocytoma. Lancet. (2005) 366:665–75. doi: 10.1016/S0140-6736(05)67139-5, PMID: 16112304

[16] Reisch N Peczkowska M Januszewicz A Neumann HPH. Pheochromocytoma: presentation, diagnosis and treatment. J Hypertens. (2006) 24:2331–9. doi: 10.1097/01.hjh.0000251887.01885.54, PMID: 17082709

[17] Zhang CX Tian Y. Adrenal collision tumor composed of adrenocortical adenoma and pheochromocytoma. Chin Med J (Engl). (2018) 131:374–5. doi: 10.4103/0366-6999.223866, PMID: 29363662 PMC5798068

[18] Thompson LDR. Pheochromocytoma of the Adrenal gland Scaled Score (PASS) to separate benign from Malignant neoplasms: a clinicopathologic and immunophenotypic study of 100 cases. Am J Surg Pathol. (2002) 26:551–66. doi: 10.1097/00000478-200205000-00002, PMID: 11979086

[19] Wachtel H Hutchens T Baraban E Schwartz LE Montone K Baloch Z. Predicting metastatic potential in pheochromocytoma and paraganglioma: A comparison of PASS and GAPP scoring systems. J Clin Endocrinol Metab. (2020) 105:e4661–4670. doi: 10.1210/clinem/dgaa608, PMID: 32877928 PMC7553245

[20] McNicol AM. Histopathology and immunohistochemistry of adrenal medullary tumors and paragangliomas. Endocr Pathol. (2006) 17:329–36. doi: 10.1007/s12022-006-0004-2, PMID: 17525481

[21] Pacak K Jochmanova I Prodanov T Yang C Merino MJ Fojo T. New syndrome of paraganglioma and somatostatinoma associated with polycythemia. J Clin Oncol. (2013) 31:1690–8. doi: 10.1200/JCO.2012.47.1912, PMID: 23509317 PMC3807138

[22] Neumann HPH Bausch B McWhinney SR Bender BU Gimm O Franke G. Germ-line mutations in nonsyndromic pheochromocytoma. N Engl J Med. (2002) 346:1459–66. doi: 10.1056/NEJMoa020152, PMID: 12000816

[23] Favier J Amar L Gimenez-Roqueplo AP. Paraganglioma and phaeochromocytoma: from genetics to personalized medicine. Nat Rev Endocrinol. (2015) 11:101–11. doi: 10.1038/nrendo.2014.188, PMID: 25385035

[24] Amodru V Taieb D Guerin C Romanet P Paladino N Brue T. MEN2-related pheochromocytoma: current state of knowledge, specific characteristics in MEN2B, and perspectives. Endocrine. (2020) 69:496–503. doi: 10.1007/s12020-020-02332-2, PMID: 32388798

[25] Mearini L Del Sordo R Costantini E Nunzi E Porena M. Adrenal oncocytic neoplasm: a systematic review. Urol Int. (2013) 91:125–33. doi: 10.1159/000345141, PMID: 23147196

[26] Tahar GT Nejib KN Sadok SS Rachid LMM. Adrenocortical oncocytoma: a case report and review of literature. J Pediatr Surg. (2008) 43:E1–3. doi: 10.1016/j.jpedsurg.2007.12.067, PMID: 18485928

[27] Gołkowski F Buziak-Bereza M Huszno B Bałdys-Waligórska A Stefańska A Budzyński A. The unique case of adrenocortical Malignant and functioning oncocytic tumour. Exp Clin Endocrinol Diabetes. (2007) 115:401–4. doi: 10.1055/s-2007-967083, PMID: 17701888

[28] Bisceglia M Ludovico O Di Mattia A Ben-Dor D Sandbank J Pasquinelli G. Adrenocortical oncocytic tumors: report of 10 cases and review of the literature. Int J Surg Pathol. (2004) 12:231–43. doi: 10.1177/106689690401200304, PMID: 15306935

[29] Mete O Erickson LA Juhlin CC de Krijger RR Sasano H Volante M. Overview of the 2022 WHO classification of adrenal cortical tumors. Endocr Pathol. (2022) 33:155–96. doi: 10.1007/s12022-022-09710-8, PMID: 35288842 PMC8920443

[30] Tallini G. Oncocytic tumours. Virchows Arch. (1998) 433:5–12. doi: 10.1007/s004280050209, PMID: 9692819

[31] Duregon E Volante M Cappia S Cuccurullo A Bisceglia M Wong DD. Oncocytic adrenocortical tumors: diagnostic algorithm and mitochondrial DNA profile in 27 cases. Am J Surg Pathol. (2011) 35:1882–93. doi: 10.1097/PAS.0b013e31822da401, PMID: 21989346

